# Unveiling Matrix Metalloproteinase 13’s Dynamic Role in Breast Cancer: A Link to Physical Changes and Prognostic Modulation

**DOI:** 10.3390/ijms26073083

**Published:** 2025-03-27

**Authors:** Xiaomeng Sun, Xiaojuan Hu

**Affiliations:** 1 Queen Mary School, Jiangxi Medical College, Nanchang University, Xuefu Avenue, Honggutan District, Nanchang 330031, China; jp4217121043@qmul.ac.uk; 2 School of Basic Medical Sciences, Jiangxi Medical College, Nanchang University, Xuefu Avenue, Honggutan District, Nanchang 330031, China

**Keywords:** MMP13, breast cancer, stiffness, structure, temperature

## Abstract

The biomechanical properties of the extracellular matrix (ECM) including its stiffness, viscoelasticity, collagen architecture, and temperature constitute critical biomechanical cues governing breast cancer progression. Matrix metalloproteinase 13 (MMP13) is an important marker of breast cancer and plays important roles in matrix remodelling and cell metastasis. Emerging evidence highlights MMP13 as a dynamic modulator of the ECM’s physical characteristics through dual mechanoregulatory mechanisms. While MMP13-mediated collagen degradation facilitates microenvironmental softening, thus promoting tumour cell invasion, paradoxically, its crosstalk with cancer-associated fibroblasts (CAFs) and tumour-associated macrophages (TAMs) drives pathological stromal stiffening via aberrant matrix deposition and crosslinking. This biomechanical duality is amplified through feedforward loops with an epithelial–mesenchymal transition (EMT) and cancer stem cell (CSC) populations, mediated by signalling axes such as TGF-β/Runx2. Intriguingly, MMP13 exhibits context-dependent mechanomodulatory effects, demonstrating anti-fibrotic activity and inhibiting the metastasis of breast cancer. At the same time, angiogenesis and increased metabolism are important mechanisms through which MMP13 promotes a temperature increase in breast cancer. Targeting the spatiotemporal regulation of MMP13’s mechanobiological functions may offer novel therapeutic strategies for disrupting the tumour–stroma vicious cycle.

## 1. Introduction

Breast cancer (BC) is the most frequently diagnosed malignancy in women that causes cancer-related death among women [1]. Approximately 90% of cancer-induced deaths are a result of metastases [2]. The primary target organs for BC metastasis are generally considered to be the bones, lungs, liver, and brain [3]. To manage and predict BC progression, it is important to detect BC cell metastases as early as possible [4]. In recent years, it has been found that the physical properties of breast cancer’s ECM—including its mechanical properties (stiffness, viscoelasticity, and plasticity) [5], extracellular matrix (ECM) structure (pore size and collagen arrangement) [6], and temperature [7]—play a critical role in regulating the behaviour of cancer cells. These findings provide powerful evidence for the diagnosis and prognosis of breast cancer. Overdiagnosis frequently leads to overtreatment, which can adversely affect patient prognosis [8]. To mitigate this issue, it is essential to identify and target key components of the tumour microenvironment and molecular markers, enabling more precise and personalised treatment strategies. Notably, the physical properties of breast cancer offer novel opportunities for achieving accurate and targeted therapeutic interventions. Interventions based on these physical properties can inhibit tumour development through multiple pathways, which is more advantageous than traditional therapy’s single mechanism of action [9]. For example, microwave-induced hyperthermia (MIH) promotes radiotherapy’s tumour cell killing via Bax-mediated cell death, boosts cellular immunity in irradiated mice, and reduces the radiotherapy-induced increase in MMP9 expression, which significantly improves lung metastasis control and overall survival in mice [10]. Thus, there is more potential for personalised treatment.

While these physical properties provide diagnostic and therapeutic opportunities, their dynamic regulation hinges on the enzymatic remodelling of the ECM, a process predominantly orchestrated by matrix metalloproteinases (MMPs) [11]. Among the MMPs, MMP13 is considered to be a key factor affecting the prognosis of breast cancer [12]. MMP13 exhibits unique characteristics that make it particularly relevant in the context of breast cancer. MMP13 is one of the few MMPs (MMP1, 8, 13, and 14) that are capable of cleaving triple-helical collagen, a critical structural component of the ECM [13]. The destruction of triple-helical collagen by MMP13 not only facilitates ECM remodelling, but also enhances the migration and metastasis of breast cancer cells [14].

MMP13 has wide-ranging substrate specificity and is more diverse in its substrate selection than other MMPs; for example, MMP2/9 are limited to the degradation of gelatine or certain collagens [15,16]. In addition, MMP13 occupies a central hub position in MMP activation networks. It not only activates key MMPs such as MMP14, 2, and 9, but is also activated by other MMPs [15,17]. Moreover, stromal MMP13 expression was found to be significantly higher in the HER2-overexpressing subtype than in the basal-like subtype (*p* = 0.016). Meanwhile, tumoural MMP13 showed a higher expression in the basal-like subtype than in the HER2-overexpressing subtype (*p* = 0.010) [18]. The combination of unique proteolytic capabilities, network centrality, and subtype-specific duality positions MMP13 as a critical node in breast cancer progression.

Despite extensive research on breast cancer biology, the functional implications of MMP13 in relation to the physical properties of breast tumours remain unexplored in the existing literature. Furthermore, the conventional paradigm attributes matrix stiffening to an imbalance between ECM deposition and degradation processes [19,20,21]. This established framework inherently excludes the possibility of concurrent MMP-mediated fluidisation and matrix stiffening within the same tumour microenvironment. Emerging evidence has demonstrated that MMP13 can simultaneously induce matrix stiffening and fluidisation—a dual regulatory mechanism that critically influences breast cancer cell invasion and migration [22]. MMP13-mediated stromal degradation creates a different microenvironment for tumour development. The mechanical properties of the ECM mediate cell–matrix interactions, resulting in cell mechanotransduction and affecting cell behaviours such as an epithelial–mesenchymal transformation (EMT), cell adhesion, diffusion, and migration, therefore resulting in different prognostic outcomes [5,23].

This study aims to introduce how the shaping of the ECM by MMP13 affects the physical properties of BC stroma, thus leading to dual prognosis results. Therefore, the expression of MMP13 in relation to these properties can be exploited for breast cancer therapy. The paradoxical association requires further study and provides new insights into breast cancer prognosis. Therefore, the existing pathways that activate or inhibit MMP13 expression in breast cancer are also discussed, with the hope of providing more ideas for the prognosis of breast cancer.

## 2. Physical Characteristics and Prognosis of Breast Cancer

### 2.1. Stiffness Is an Indicator of Poor Prognosis in Breast Cancer

Solid tumours exhibit greater stiffness compared to healthy tissue. This characteristic is linked to an overall elevated lifetime risk of malignancy [24,25], which has been utilised for the detection of breast cancer, either through physical palpation or using imaging modalities such as magnetic resonance imaging, computerised tomography, and elastography [26,27]. The deposition of the matrix and pathological crosslinking of collagen are the primary factors contributing to the increased stiffness of the ECM in breast cancer [28]. Moreover, other cells within the tumour microenvironment (TME), such as cancer-associated fibroblasts (CAFs) and tumour-associated macrophages (TAMs), along with ECM remodelling, also contribute to enhancing stiffness [29].

Elevated ECM stiffness promotes focal adhesion formation and augments cytoskeletal contractility, thereby potentiating the growth factor receptor-mediated activation of ERK and PI3K signalling pathways in tumour cells [28]; the ERK and PI3K signalling pathways regulate diverse cellular processes, including proliferation, apoptosis, and metabolic reprogramming, with their activation driving breast cancer progression and malignancy [30]. Furthermore, the stiffness of the ECM can trigger a breast cancer EMT through mechanical transduction [29]. During an EMT, there is a downregulation of proteins crucial for maintaining a polarised epithelium, such as occludin, E-cadherin, and claudins, accompanied by an upregulation of mesenchymal proteins [31]. These changes result in a decrease in cell–cell adhesion and an increase in motility, and the tumour cells lose polarity, increasing their aggressiveness. Consequently, tissue integrity is impaired [32].

A stiff ECM is often linked to a poor prognosis for breast cancer. The stiff matrix induces the expression of pro-metastatic and neurotrophic genes via the integrin β1-FAK-YAP signal pathway, which finally promotes perineural invasion (PNI) in BRCA [33]. Substrates with intermediate stiffness are most effective in regulating cell drug resistance, and the proportion of dead cells is significantly decreased. This is because the expression of integrin-linked kinase (ILK) is highest on intermediate-stiffness substrates. ILK can mediate the activity and nuclear translocation of Yes-associated protein (YAP), thereby regulating stiffness-dependent drug resistance [34]. Matrix stiffness influences the transition from a normal phenotype to a more proliferative and invasive one by controlling the activation and expression of integrins [5,35]. Triple-negative breast cancer (TNBC)—the most aggressive molecular subtype of breast cancer—is characterised by the absence of oestrogen receptor (ER) and progesterone receptor (PR) expression, as well as a lack of epidermal growth factor receptor 2 (ERBB2) gene amplification or ERBB2 protein overexpression [36]. Compared to other breast cancer subtypes, TNBC exhibits the highest enrichment of cancer stem cells (CSCs) [37]. As cancer progresses, tissue stiffness may be an inherent response of CSCs to optimise the growth of cancer cells. Breast cancer stem cells (BCSCs) drive angiogenesis through dual mechanisms: direct differentiation into endothelial cells and the paracrine secretion of pro-angiogenic factors [38]. BCSCs express drug efflux transporters and multi-drug resistance genes, which endow them with resistance to conventional chemotherapeutic drugs [39]. Matrix stiffness can influence cell contractility (related to the cytoskeleton) and enzymes involved in cell growth and differentiation, such as Rho and ERK [40]. Collectively, these factors contribute to the metastasis, recurrence, and therapy resistance of breast cancer [38].

### 2.2. Mechanical and Structural Characteristics Influence Breast Cancer Metastasis

Cell migration is influenced by various ECM properties, including the pore size, fibre arrangement, and other characteristics. If the pore diameter of the ECM exceeds approximately 3 μm, cells can migrate by squeezing through the pores [38,41,42]. In contrast, ECM structures with reduced pore dimensions necessitate alternative migration strategies. Cells may employ proteolytic enzymes to degrade ECM components [42,43,44] or utilise mechanical forces to create migration pathways, provided that the ECM demonstrates adequate mechanical plasticity [5,23]. This mechanical plasticity is closely associated with viscoelasticity, which describes materials exhibiting both elastic solid and viscous liquid behaviours, characterised by time-dependent mechanical responses such as creep or stress relaxation [5]. Mechanical stress induces the rupture of weak crosslinks within or between fibres, resulting in fibre elongation, reorientation, and energy dissipation through viscous flow [45,46,47]. These irreversible fibre movements become stabilised through the reformation of weak crosslinks, leading to permanent or plastic deformation. Consequently, the rupture of weak bonds that enable viscoelastic creep and stress relaxation simultaneously contributes to plastic deformation, thereby establishing a connection between viscoelasticity and plasticity [5].

These properties are intrinsically linked to matrix remodelling processes. The cellular remodelling of viscoplastic matrices directly influences their pore dimensions [48], while matrix degradation alters their viscoelastic characteristics [49]. Furthermore, modifications in matrix architecture likely impact both viscoplasticity and degradability simultaneously [23]. Such remodelling processes significantly influence cell migration by modifying the structural organisation and fibre alignment within the matrix [5,50]. Within the interstitial stroma, collagen fibrils serve as preferential pathways for cellular migration, facilitating cancer cell dissemination along their longitudinal axes [13]. Conversely, the dense meshwork of stromal collagen fibrils and the desmoplastic capsule surrounding tumour masses analogous to the type IV collagen network in basement membranes (BMs) create substantial barriers to tumour cell infiltration [42,51]. This structural dichotomy highlights the critical role of matrix organisation in regulating cellular movement and tumour progression.

Research evidence indicates that appropriate levels of mechanical plasticity and viscoelasticity are critical regulators of tumour cell motility [23,52]. Specifically, the mechanical plasticity of the extracellular matrix facilitates the development of collagen-based migration tracks, which serve as structural pathways for carcinoma cells during their dissemination from primary tumour sites [5]. The mechanism underlying enhanced cell migration involves cellular adaptation to high-resistance microenvironments, which stimulates the assembly of dense actin cytoskeletal networks. This structural reorganisation facilitates the localised accumulation of ion transporters that functionally coordinate with aquaporins to mediate the cellular water uptake. The resultant osmotic expansion elevates membrane tension, triggering the activation of TRPV4 channels and subsequent calcium influx, a mechanosensitive signalling cascade that collectively drives migratory behaviour [52]. Furthermore, the viscoelastic properties of tissues have a significant diagnostic value in breast cancer detection [53]. Leveraging this biological characteristic, shear wave elastography (SWE) has emerged as a valuable non-invasive imaging technique for breast cancer diagnosis. This innovative modality utilises tissue viscoelasticity as a diagnostic parameter, providing clinically relevant information for tumour detection and characterisation [54,55].

Tumour-associated collagen typically exhibits distinct alignment patterns and anisotropic properties, enabling the classification of collagen organisation through the Tumour-Associated Collagen Signature (TACS) system, which serves as a prognostic indicator for breast cancer patients [56]. TACS-1 means that there is an accumulation of collagen in the adjacent tissue, progressive TACS-3 means that the collagen fibres are oriented perpendicular to the tumour border, and TACS-2 is an intermediate process of tumour development and tends to inhibit tumour invasion [57].

### 2.3. Thermal Properties: Increased Angiogenesis and Metabolism Lead to High Temperature

Breast cancer, being the most prevalent oncological diagnosis, exhibits distinct thermal characteristics compared to normal tissue, with the temperature differentials reaching up to 3.5 °C in affected regions [7]. Clinical observations demonstrate that patients presenting with elevated tumour temperatures (hot tumours) experience significantly reduced disease-free and disease-specific survival rates compared to those with lower temperature malignancies (cold tumours) [7]. This thermal disparity stems from the characteristic hypermetabolic state of malignant tumours, coupled with their extensive vascular network, which collectively contributes to localised temperature elevation [58,59]. These pathophysiological features form the foundation for medical thermography applications in breast cancer detection and the identification of precancerous cellular proliferation [59]. In response to hypoxic conditions and other microenvironmental stimuli, tumour cells initiate the secretion of multiple signalling molecules, including hypoxia-inducible factor (HIF) [58], vascular endothelial growth factors (VEGFs), and angiopoietins, which collectively drive angiogenesis. VEGFs’ expression is regulated through multiple pathways, including transforming growth factor-β (TGF-β) signalling [60] and the tumour necrosis factor-α (TNF-α)/nuclear factor-κB (NF-κB) axis [61]. The resulting neovasculature within the tumour microenvironment (TME) exhibits structural abnormalities, characterised by disorganisation and increased vascular permeability. These pathological alterations exacerbate intratumoural hypoxia, facilitate metastatic dissemination, and simultaneously impair the drug delivery efficiency while suppressing antitumour immune responses [58,62].

## 3. MMP13 Is the Key to Regulating These Physical Properties

### 3.1. The Background of MMP13

An important factor that regulates the above physical properties is MMPs, which are a group of calcium-dependent, zinc-containing endopeptidases that can be found in the ECM [63,64,65]. MMPs play a pivotal role in the degradation of the ECM and BMs within tumours [11] and can be released by CAFs [66], primary tumour cells, and immune cells within the TME [67]. Increasing evidence indicates that MMP expression is elevated in breast cancer, promoting EMT and ECM remodelling, as well as mediating angiogenesis; thus, the microenvironment is changed, which facilitates cancer cell invasion and metastasis [11,68].

Among the 28 structurally related MMPs currently known [63,64,65], MMP13, also named collagenase-3 [69], has been proven to be linked to malignancy, affecting cell proliferation and migration [25]. Normally, the level of MMP13 is tightly regulated [65]; MT1-MMP (MMP14) [70], MMP3, MMP2, trypsin-2 [65], and plasmin [71] can activate pro-MMP13 to MMP13. High MMP13 is commonly detected in breast tumours accompanied by a positive test for cancer cells in the lymph nodes [72] as well as at the tumour–bone (TB) interface [17]. Elevated MMP13 expression levels were significantly correlated with decreased distant metastasis-free survival, particularly in oestrogen receptor-negative breast cancer cases. Patients with high MMP13 expression demonstrated a 2.25-fold increased risk of metastasis (95% confidence interval: 1.32–3.84; *p* = 0.0023) compared to those with low expression levels [73].

### 3.2. Effect of MMP13 on Tumour Stiffness

MMP13 directly alters the mechanical properties of the tumour microenvironment by degrading collagen and other matrix components in the ECM. The main function of MMP13 is ECM remodelling [3]. Furthermore, collagen reorganisation can be driven by MMP13 to create a more suitable environment for breast cancer cell development [66]. MMP13 is more inclined to degrade collagen I-III. In addition, gelatine, perlecan, large tenascin C, fibronectin, plasminogen, aggrecan, fibrillin-1, and osteonectin are substrates of MMP13 [63,65]. The structure of collagen is destroyed, and the stiffness of the ECM is reduced [74]. MMP13 reduces the stiffness of the ECM through degradation, which provides a softer microenvironment and is beneficial for the survival of disseminated breast cancer cells at secondary sites [75]. When breast cancer cells were cultured on soft matrices simulating metastatic microenvironment conditions, studies demonstrated that these malignant cells exhibited prolonged dormancy, evading the cytotoxic effects of chemotherapeutic agents [76]. Additionally, a compliant extracellular matrix promotes chemoresistance development through autophagy enhancement mechanisms, thereby contributing to the complexity of managing metastatic breast cancer [75,76].

However, the effect of MMP13 on the stiffness of the ECM is dynamic. Although the ECM is softened in the short-term, its stiffness increases in the long-term. The ECM’s degradation by MMP13 can release sequestered growth factors, such as fibroblast growth factors (FGFs) and transforming growth factors (TGFs), which aid tumour cell proliferation and are also key to upregulating matrix stiffness [77,78]. These factors promote collagen crosslinking by activating stromal cells such as CAFs [79] and upregulating lysyl oxidases (LOXs) for collagen (type I and type III) deposition and rearrangement; thus, the ECM gradually transforms into a dense fibrous tumour stroma [80,81]. This dynamic balance may explain the spatiotemporal heterogeneity of tumour mechanics: softened metastases favour cell dormancy, while hardened primary lesions drive aggressive growth.

Beyond its biomechanical effects on ECM compliance, MMP13 orchestrates epithelial–mesenchymal plasticity through mechanotransduction pathways. MMP13 not only affects the tumour microenvironment through ECM remodelling, but also indirectly changes the physical properties of tumours by promoting an EMT, reducing intercellular adhesion, and making tumour cells more loosely arranged [2,82]. When analysing gene expression during an EMT, the researchers found that MMP13 mRNA levels were upregulated at least 16.6-fold in mesenchymal EpRasXT cells [83]. While MMP13 promotes the EMT, breast cancer cells produce more MMP13 during an EMT, therefore creating positive feedback [84].

The MMP13 gene promoter comprises numerous TF binding sites [85,86], including the TATA box region (TATAAA) [69,86], the AP-1 site [87], the ETS/PEA-3 site [88], and the RD sites, also named Cbfa1 (core binding factor 1) [89,90] or OSE2 (osteoblast-specific element 2) sites [91]. The activation of these sites promotes MMP13 gene expression. Adjacent sequences of AP-1 sites are important for maximum AP-1 transcriptional activation [92]. The PEA-3/AP-1 combination responds to growth factors, tumour promoters, and oncogenes [85]. The 5-GGAA-3 sequence of the PEA-3 site cooperates with the AP-1 site in regulating MMP13 gene transcription [93]. The expression of MMP13 in BC is triggered by the ETS variant transcription factor 4 (ETV4) binding to the AP-1 region [94].

The expression of MMP13 is regulated by a variety of signalling pathways, which have been studied in the MDA-MB-231 cell line. In response to TGF-β1, the expression of Cbfa1/Runx2 increases; it interacts with the MMP13 promoter’s distal RD site and is stabilised by TGF-β1/Smad3 signalling [92]. Further experiments in MDA-MB-231 cells revealed that knocking down ATF-3 expression reduced its binding to the Runx2 promoter [95]. Studies in the same cell line also showed that Codonolactone (CLT) can downregulate the binding activity of Runx2, therefore inhibiting Runx2’s binding to the MMP13 promoter [96]. Importantly, the MDA-MB-231 system was used to establish the essential role of the Runx2–ABL complex in MMP13 expression. The tyrosine kinase ABL directly phosphorylates and activates Runx2 via its SH2 domain in a manner that relies on its kinase activity, and ABL–Runx2 is also formed [97].

Researchers have found that, in breast cell lines like MDA-MB 231, microRNAs such as miR-203 and miR-135 [98,99,100] can inhibit the EMT and MMP13 expression by targeting RUNX2 [98]. Emerging evidence suggests that RUNX2 potentially modulates the EMT via secretory factors like MMP1 and critical signalling cascades including TGF-β and Wnt pathways. This EMT activation subsequently governs BCSC dynamics, ultimately culminating in therapeutic resistance development in breast malignancies [101]. CADD522 is a small molecule that is found to inhibit the DNA binding of Runx2 [20]. Molecularly, Runx2, ATF-3, and MMP13 were downregulated in MDA-MB 231 cells upon the forced expression of miR-4638-3p [21].

The proteolytic processing of the ECM by MMPs exposes hidden binding sites that are functionally associated with cell survival, thereby facilitating integrin-mediated tumour cell–ECM interactions [102]. The mechanical stretching TGF-β signalling pathway in normal myoepithelial cells induces a ductal carcinoma in situ (DCIS) phenotype associated with integrin-β6 expression, thereby affecting the expression of proteases such as MMP13 and promoting the invasion behaviour of tumour cells [103]. Matrix stiffness promotes integrin clustering, which subsequently amplifies ERK signalling pathway activation and enhances ROCK-dependent cytoskeletal contractility along with focal adhesion formation, ultimately driving tumour progression toward malignant phenotypes [104]. A rigid ECM activates the mechanosensitive regulators YAP1 and TAZ, which translocate to the nucleus, form complexes with TEAD, and modulate gene expression programmes controlling cell motility, proliferation, survival, and stem cell properties [105,106,107]. The nuclear accumulation of YAP/TAZ can modify E/N cadherin and vimentin expression in a stiffness-dependent manner, driving the EMT [108,109,110]. A rigid ECM is also conducive to the enrichment of CSCs [111]; the plasticity of CSCs causes TNBC cells to have higher tumourigenic and metastatic potential and adapt to and resist traditional anticancer therapies, resulting in tumour progression and recurrence [38]. Therefore, it is speculated that MMP13 may indirectly affect the enrichment of CSCs in TNBC by influencing the state of the ECM and then participating in the development of TNBC. These findings suggest that targeting MMP13 and its regulatory network may provide new strategies for TNBC treatment.

### 3.3. Effect of MMP13 on Tumour Stromal Structure

The ECM is a three-dimensional network that supports cell activity and interactions [97,112]. Under the action of MMP13, the structure and plasticity of the ECM are changed, creating conditions for the metastasis of breast cancer-related cells [65]. Typically, tumours occur in situ with the basement membrane preventing them from invasion and metastasis, whereas an absence or breach of the BM denotes invasion [113].

MMP13 belongs to the collagenases, which are often key to breaking down these barriers [114]. As MMP13 can activate MMP9 by cleaving the inactive pro-MMP9 forms [17], its action on MMP9 activation facilitates tumour cell intravasation into vascular and lymphatic systems at primary tumour sites. At the same time, it can also promote the extravasation (the process of tumour cells moving out of blood/lymphatic vessels) of invaded tumour cells from blood/lymphatic vessels in remote organs [65]. However, Perry et al. found that the MMP13-induced alteration of collagen orientation can hinder tumour cell migration. In mice without MMP13 KO, longer and thicker collagen I fibres are more frequently oriented parallel to the breast tumour–host boundary [115]. The straight, “taut” fibres parallel to the tumour boundary show a TACS-2 collagen configuration, which has always been associated with regions of decreased tumour invasiveness [81]. Perry et al. [115] also found that the presence of MMP13 reduced the metastasis of a primary breast cancer tumour to the lungs. In addition, the study of Ruoqing et al. [116] showed that in a mouse model of primary breast tumours, MMP13 expression was markedly reduced in premetastatic lung tissues relative to control samples. This is different from the conventional findings that gremlin-1 (GREM1) can facilitate breast cancer pulmonary metastasis via a signal transducer and activator of transcription (STAT) 3-mediated MMP13 regulation [73]. The observed differences in outcomes between the inhibitory effects of MMP13 on breast cancer lung metastasis in mouse models and its promotion of metastasis in human breast cancer warrant further investigation.

MMP13 is an important protease for remodelling the ECM, and its degradation of ECM-related substances has shaped different plastic and viscoelastic characteristics of the ECM. Inna et al. [117] showed that intact ECM had the highest viscosity (G″~1.75 kPa), while the ECM viscosity decreased (G″~0.6 kPa) after MMP13’s action. Also, more cells adhered to degraded ECM than to natural ECM. The TRPV4-channel-mediated Ca^2+^ flow is a signalling pathway necessary for IL-1 to induce MMP13 expression [118]. High-resistance environments induce TRPV4 expression, accelerate the endogenous Ca^2+^ flow, and encourage cell migration [52]. Putting the evidence together, high resistance triggers the release of MMP13, which encourages cell metastasis. However, due to the lack of relevant studies, conclusions cannot be made, and more studies are needed to reveal the association between MMP13 and viscoelasticity regarding tumour migration.

### 3.4. Effect of MMP13 on Tumour Stromal Temperature

MMP13 is positively associated with macrophages [119]. TAM-derived IL-1β induces the expression of pro-tumourigenic cytokines, including IL-6, TNF-α, and TGF-β [120]. During initial tumourigenesis, immune-cell-secreted IL-1β triggers NF-κB activation in cancer-associated fibroblasts, stimulating their secretion of pro-tumour inflammatory mediators [28]. Inflammatory cells can secrete various pro-angiogenic factors such as IL-8, which can stimulate the expression of VEGF and MMP [121]. VEGF plays an important role in tumour angiogenesis [58]. Through Matrigel plug evaluation, it was found that a high expression of VEGF would increase the microvessel density (MVD) [122]. Inflammation and an increased blood vessel density can cause breast cancer tumour temperatures to rise.

VEGFs are released by a variety of cells in the TME, such as TAMs [123]. MMPs primarily mediate extracellular matrix degradation, facilitating endothelial cell migration and neovascularisation. MMP13 specifically degrades interstitial collagens, initiating ECM remodelling and stimulating angiogenesis in a chicken embryo model [124].

Furthermore, it enhances tissue infiltration and invasion, potentially contributing to metastatic progression [125,126]. Within the tumour microenvironment, MMP overexpression compromises extracellular matrix integrity, facilitating collective cancer cell migration through tortuous and leaky vessels to distant sites [127]. Angiogenesis involves a coordinated regulation of some vascular growth factors, such as basic fibroblast growth factor (bFGF/FGF2) and transforming growth factor TGF-β1 [121], which can be released through ECM degradation mediated by MMP13 [77,78]. Furthermore, studies in the 4T1 cell line and mesenchymal stem cells (MSCs) show that inflammatory signals, such as TNF-α, can induce the expression of CXCR2 ligands, leading to the recruitment of neutrophils to the tumour site. These processes are associated with the increased expression of metastasis-related genes, including MMP13 [128]. This positive feedback loop, driven by elevated MMP13 levels, further promotes TAM infiltration and angiogenesis, and ultimately contributes to the rise in breast cancer tumour temperatures.

**Figure 1:**
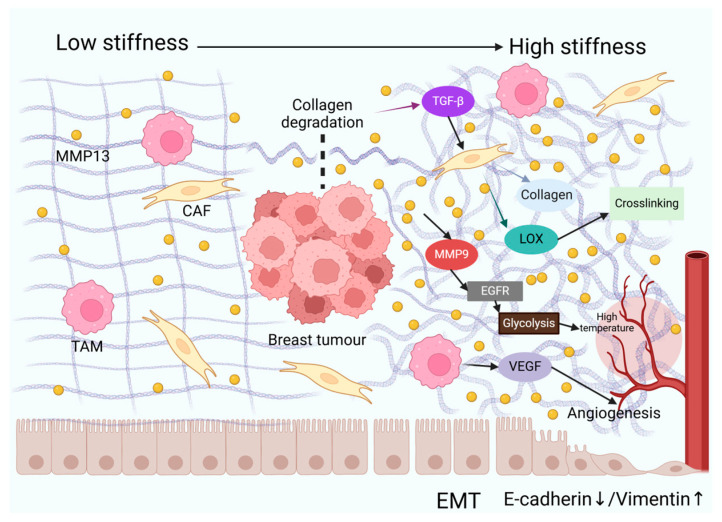
MMP13 orchestrates dynamic remodelling of the extracellular matrix (ECM)’s physical properties. In breast cancer, elevated levels of MMP13 in the extracellular matrix (ECM) degrade collagen, releasing growth factors such as TGF-β, which promote epithelial–mesenchymal transition (EMT) in tumour cells (characterised by downregulation of E-cadherin and upregulation of vimentin), thereby enhancing invasive capacity. MMP13 activates cancer-associated fibroblasts (CAFs) to secrete collagen and lysyl oxidase (LOX), promoting collagen crosslinking to form a dense stromal matrix that increases tumour stiffness. MMP13-activated MMP9 triggers the EGFR signalling pathway, enhancing glycolysis and elevating tumour temperature. Tumour-associated macrophages (TAMs) secrete vascular endothelial growth factor (VEGF), synergising with MMP13 to promote angiogenesis and increase local temperature.

Furthermore, activated MMP9 regulates the process of angiogenesis [129] and the EGFR pathway [11]. The activation of the EGFR pathway can enhance glycolysis in TNBC cells. In a study, after treating TNBC cells with the EGFR activator NSC228155, the mRNA expression of glycolysis-related molecules (such as PFKL, GLUT1, HK2, and PKM2) increased. The glucose uptake, lactic acid production, and ATP levels were also significantly increased [130]. Increased metabolism raises the local temperature of breast cancer. In summary, MMP13 dynamically remodels the physical properties of the ECM, including the stiffness, architecture, and thermodynamic characteristics. These multifaceted mechanisms collectively drive the malignant progression of breast cancer (Figure 1).

## 4. MMP13 as a Central Regulator in Breast Cancer TME: Crosstalk with Cells to Modulate Physical Properties

### 4.1. MMP13 in TAM-Mediated Breast Cancer Progression

Having established MMP13’s multifaceted roles in biomechanical modulation, we next dissect its cellular crosstalk within the breast tumour microenvironment. MMP13 promotes TAM infiltration [128]. TAMs release factors including VEGF, MMPs, platelet-derived growth factor (PDGF), and angiopoietin-1 [131,132]. M2 macrophages secrete VEGF and MMPs, leading to angiogenesis and ECM remodelling [123]. TAMs secrete various soluble factors that induce ECM deposition, thereby stiffening the extracellular matrix [25].

TAMs also release several other factors contributing to the expression of downstream pro-tumour cytokines, therefore promoting the physical properties of BC ECM changes [123]. It is worth noting that MMP13 shows dual effects in microenvironments. This duality can be attributed to several factors, including the dynamic nature of the ECM, the composition and organisation of ECM components, and also the specific cellular sources of MMP13. MMP13 acts as a collagenase that degrades collagen and shows anti-fibrotic properties in the liver [133,134], ovaries [135], and lungs [136]. However, when the ECM is degraded and ECM remodelling is promoted in the tumour, MMP13 also plays a role in promoting breast tumour fibrosis in the long-term [77,78]. This difference may be linked to variations in the ECM composition and organisation. It is worth noting that inactive MMPs may be observed in the process of fibrosis [75]. For example, when collagen is crosslinked, MMPs cannot break down the collagen, thus increasing the matrix stiffness [137].

The duality of MMP13 is also closely tied to its cellular sources. M2 TAMs, which are generally pro-tumourigenic, release MMPs that promote breast cancer progression [123]. In contrast, M1 TAMs, which are considered tumour-suppressive [123], secrete MMP13 and exert anti-stiffness effects in breast cancer. Dinesh et al. [138] showed that, within the breast cancer TME, Slit2-activated macrophages are highly phagocytic and polarised toward the antitumour M1 phenotype, and secrete MMP13 into the ECM, which inhibits fibrosis.

Furthermore, M1 macrophages activate the STAT3/NF-κB signalling pathway in breast cancer cells through inflammatory cytokines (IL-6, TNF-α, and IL-1β), which then triggers the activation of the lin-28b–let-7–hmga2 axis, causing an EMT and inducing the formation of CSCs [137]. Thus, while MMP13 and M1 TAMs can have anti-fibrotic and tumour-suppressive effects, their roles in the TME are complex and context-dependent.

### 4.2. MMP13 in CAF-Driven Breast Cancer Progression

In breast cancer, MMP13 can be secreted by CAFs and the primary tumour [12], also promoting BM degradation and tumour cell migration [29]. The pro-oncogenic transcription factor STAT3 forms a positive feedback loop with some of the inflammatory cytokines, including IL-6, and promotes MMP13 expression in murine breast cancer CAFs; furthermore, MMP13 plays an important role in maintaining the function of CAFs [139]. A positive expression of MMP13 in CAFs was associated with an enhanced OR (odds ratio) for regional metastasis [140]. CAFs promote aggressive phenotypes of breast cancer cells through an EMT induced by paracrine TGF-β1, and the TGF-β/Smad signalling pathway can also be activated by CAFs [141]. TGF-β usually promotes tumourigenesis by enhancing cellular transformation, an EMT, invasion, and metastasis [60].

**Figure 2:**
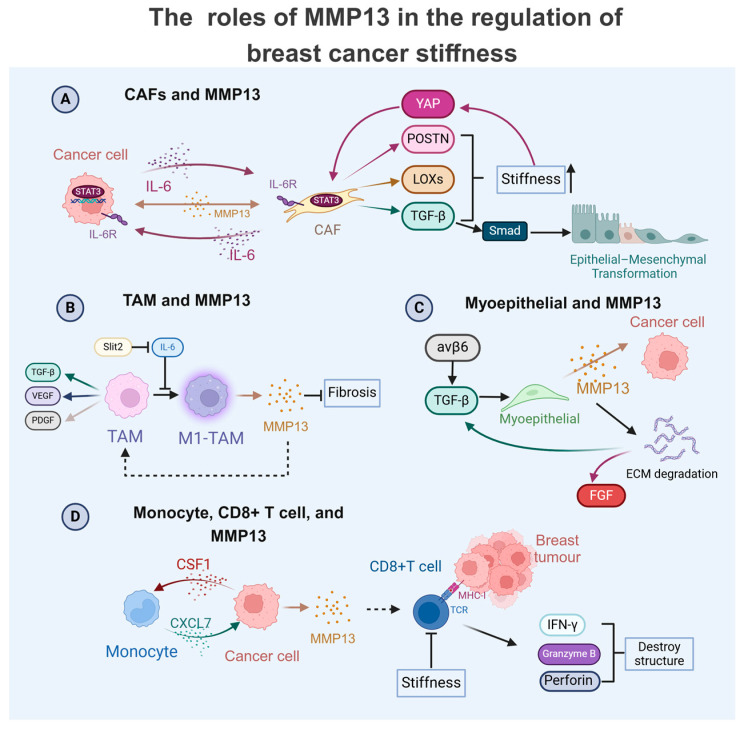
Multifaceted roles of MMP13 in modulating the breast cancer matrix’s physical properties. (**A**) Tumour-derived IL-6 activates cancer-associated fibroblasts (CAFs) through the STAT3-mediated transcriptional upregulation of MMP13. This protease amplifies CAF activity, creating a stiffness-enhancing feedback loop through multiple mechanisms: Matrix hardening induces YAP activation, promoting periostin (POSTN) secretion. The concomitant release of LOX enzymes and TGF-β further reinforces the matrix’s rigidity. A TGF-β/Smad signalling cascade drives epithelial–mesenchymal transition (EMT) in tumour cells; (**B**) Slit2 mediates the M1 polarisation of tumour-associated macrophages (M1-TAMs), triggering MMP13 secretion. This metalloproteinase exhibits dual functionality by reducing tumour fibrosis while facilitating TAM infiltration, which can release factors like TGF-β, VEGF, and PDGF, changing the physical properties. (**C**) Integrin-β6 signalling in myoepithelial cells stimulates MMP13 release, which directly impacts cancer cells through the following: (1) the ECM degradation-mediated liberation of growth factors (FGF/TGF-β); (2) the paracrine modulation of tumour cell behaviour. (**D**) CSF1 from tumour cells induces monocyte-derived CXCL7 production, creating an autocrine loop that upregulates MMP secretion in cancer cells. MMP13 enhances antitumour immunity by facilitating CD8+ T cell infiltration, promoting the release of cytotoxic effectors (IFN-γ, granzyme B, and perforin). Notably, ECM stiffening impairs CD8+ T cell functionality.

CAFs play a significant role in inducing tumour stiffness [134]. They synthesise and remodel the interstitial matrix (Figure 2), secrete collagen procollagen molecules, and overexpress lysyl oxidases (LOXs) involved in collagen (types I and III) deposition and rearrangement; the normal ECM gradually transforms into a dense fibrous tumour stroma [80,81]. MMPs can collaborate with LOXs to facilitate collagen maturation, and they regulate the expression and activity of soluble factors such as TGF-β that regulate the tumour cell behaviour [142,143,144,145,146]. Increased matrix stiffness activates YAP, promoting the secretion of periostin (POSTN) in CAFs, which in turn augments the matrix rigidity of mammary glands and breast tumour tissues by facilitating collagen crosslinking [147]. Beyond MMP13 expression, CAFs also influence tumour stiffness and development by secreting other factors. In addition to their effects on the ECM and secretion of soluble factors, CAFs can also modulate tumour cell behaviour through direct cell–cell contact. Through heterophilic E-cadherin/N-cadherin interactions, CAFs generate mechanical tension on tumour cells by recruiting cytoskeletal regulators including α-actinin, vinculin, nectin-1/2, and afadin. This molecular complex maintains cellular polarity while facilitating tumour cell detachment and subsequent invasion [28].

### 4.3. MMP13 with Immune and Stromal Cells in Breast Cancer Progression

In addition to CAFs and TAMs, MMP13 promotes other cells in the TME to regulate the malignancy of breast cancer (Figure 2). MMP13 is also positively correlated with CD8+T infiltration [119], which specifically identifies tumour-associated antigenic peptides presented on cell surfaces and mediates cytotoxic effects through the release of IFN-γ, granzyme B, and perforin, ultimately inducing tumour cell lysis. Unexpectedly, increased CD8+ T cell infiltration demonstrates a significant correlation with improved clinical outcomes in TNBC patients [148]. However, the function of CD8+ T cells is impaired by the stiffness of the fibrotic tumour microenvironment, which is not conducive to its antitumour immune function [149].

Breast cancer cells secrete colony-stimulating factor 1 (CSF1) to induce the monocytic expression and secretion of CXC motif chemokine ligand 7 (CXCL7) in the TME [150]. CXCL7 further stimulates the secretion of MMPs in breast cancer cells, therefore promoting ECM remodelling and also enhancing breast cancer cells’ metastasis and invasion [150]. This positive feedback loop significantly aggravates the malignant progression of the tumour.

Ductal carcinoma in situ (DCIS) is a non-obligate precursor of invasive breast cancer [151]. A high level of myoepithelial integrin-β6 is a biomarker indicative of high-risk DCIS in a heterocellular spheroid model of DCIS, which promotes the MMP13-related myoepithelial-led invasion of luminal cells through the TGF-β-dependent stimulation of EP300, which epigenetically modulates myoepithelial-derived MMP13 expression [151]. Furthermore, the mechanical stretching TGF-β signalling pathway in normal myoepithelial cells induces a DCIS phenotype associated with integrin-β6 expression, thereby affecting the expression of proteases such as MMP13 and promoting the invasion behaviour of tumour cells [103].

## 5. Conclusions

In this review, the recent advances made in understanding the roles of MMP13 in regulating the physical properties of breast cancer regarding stiffness, plasticity, viscoelasticity, collagen alignment, and temperature have been discussed. The main function of MMP13 is matrix degradation and matrix remodelling, and this process is dynamic. An overly rigid matrix or excessive matrix degradation can promote the progression of breast cancer. It also plays an important role in promoting tumour metastasis and angiogenesis. MMP13 also exhibits several functions that inhibit tumour progression, such as inhibiting fibrosis and BC lung metastasis. As a potential target, MMP13 has been proven to act as a downstream target to inhibit breast cancer. For instance, Kim et al. [69] demonstrated that sauchinone inhibits breast cancer progression by suppressing the Akt–CREB–MMP13 signalling pathway. Additionally, the silencing of the lncRNA PART1 reduced the expression of MMP13, thereby inhibiting the development of breast cancer [152]. However, MMP13 inhibitors are not yet in clinical use [153]. Therefore, the role of MMP13 in breast cancer needs to be further studied. Given the dual nature of MMP13, how to take advantage of the different effects of MMP13 under different conditions is a possible future research direction.

## Data Availability

Not applicable.

## Abbreviations

BC	Breast cancer
ECM	Extracellular matrix
MMP13	Matrix metalloproteinase 13
TME	Tumour microenvironment
CAFs	Cancer-associated fibroblasts
TAMs	Tumour-associated macrophages
EMT	Epithelial–mesenchymal transition
CSCs	Cancer stem cells
TGF-β	Transforming growth factor-β
IL-6	Interleukin-6
YAP	Yes-associated protein
POSTN	Periostin
LOXs	Lysyl oxidase
STAT3	Signal transducer and activator of transcription 3
FGF	Fibroblast growth factor
DCIS	Ductal carcinoma in situ
IFN-γ	Interferon-γ
CSF1	Colony-stimulating factor 1
CXCL	CXC motif chemokine ligand
NF-κB	Nuclear factor κB
TNF-α	Tumour necrosis factor-α
VEGF	Vascular endothelial growth factor
HIF	Hypoxia inducible factor
AP-1	Activator protein-1
ETS/PEA-3	E26 transformation-specific/polyomavirus enhancer activator-3
ETV4	ETS variant transcription factor 4
TB	Tumour bone
ER	Oestrogen receptor
PNI	Perineural invasion
ILK	Integrin-linked kinase
TNBC	Triple-negative breast cancer
BCSCs	Breast cancer stem cells
SWE	Shear wave elastography
TACS	Tumour-associated collagen signature
BM	Basement membrane
STAT3	Signal transducer and activator of transcription 3
PDGF	Platelet-derived growth factor
bFGF/FGF2	Basic fibroblast growth factor
NF1	Neurofibromin 1
ATF3	Activating transcription factor 3
NFATC2	Nuclear factor of activated T cells 2
Runx2	Runt-related transcription factor 2
ABL	Abelson tyrosine kinase
CLT	Codonolactone
RKIP	Raf kinase inhibitor protein
STEAP1	Six transmembrane epithelial antigen of the prostate 1
MSCs	Mesenchymal stem cells
